# Agglomerated novel spray-dried lactose-leucine tailored as a carrier to enhance the aerosolization performance of salbutamol sulfate from DPI formulations

**DOI:** 10.1007/s13346-017-0462-8

**Published:** 2017-12-19

**Authors:** Carlos Molina, Waseem Kaialy, Qiao Chen, Daniel Commandeur, Ali Nokhodchi

**Affiliations:** 1 Pharmaceutics Research Laboratory, School of Life Sciences, University of Sussex, Brighton, UK; 2 School of Pharmacy, Faculty of Science and Engineering, University of Wolverhampton, Wolverhampton, UK; 3 Drug Applied Research Center and Faculty of Pharmacy, Tabriz Medical Sciences University, Tabriz, Iran

**Keywords:** Dry powder inhaler, Spray-drying, Agglomeration, Salbutamol sulfate, Leucine

## Abstract

Spray-drying allows to modify the physicochemical/mechanical properties of particles along with their morphology. In the present study, _L_-leucine with varying concentrations (0.1, 0.5, 1, 5, and 10% *w*/*v*) were incorporated into lactose monohydrate solution for spray-drying to enhance the aerosolization performance of dry powder inhalers containing spray-dried lactose-leucine and salbutamol sulfate. The prepared spray-dried lactose-leucine carriers were analyzed using laser diffraction (particle size), differential scanning calorimetry (thermal behavior), scanning electron microscopy (morphology), powder X-ray diffraction (crystallinity), Fourier transform infrared spectroscopy (interaction at molecular level), and in vitro aerosolization performance (deposition). The results showed that the efficacy of salbutamol sulfate’s aerosolization performance was, in part, due to the introduction of _L_-leucine in the carrier, prior to being spray-dried, accounting for an increase in the fine particle fraction (FPF) of salbutamol sulfate from spray-dried lactose-leucine (0.5% leucine) in comparison to all other carriers. It was shown that all of the spray-dried carriers were spherical in their morphology with some agglomerates and contained a mixture of amorphous, α-lactose, and β-lactose. It was also interesting to note that spray-dried lactose-leucine particles were agglomerated during the spray-drying process to make coarse particles (volume mean diameter of 79 to 87 μm) suitable as a carrier in DPI formulations.

## Introduction

Using the pulmonary tract as an avenue for the administration of therapeutic agents to tackle chronic obstructive pulmonary disease (COPD), asthma, bronchitis, and cystic fibrosis are just a few examples of how the route of inhalation is used in a systematic fashion for drug delivery [1]. Tackling the disadvantages faced by dry powder inhalers (DPIs) begins with selecting a carrier such that it provides a good delivery of the active pharmaceutical ingredient (API) of interest [2–7]. The chosen carrier does not only need to be safe and cost-effective, and pass strict pharmacopeia guidelines, but, more importantly, it needs to be selected in such a manner that it adheres to chemical properties that will not interfere with the drug delivery process and that complement the API chosen [5, 8–13].

To date, lactose is the most commonly documented carrier in the pharmaceutical industry due to being highly stable, adhering to good flow properties, and being widely accepted as a safe excipient for use [13]. Although other carriers such as mannitol [14] and sorbitol [15] as well as other sugars [15] have been suggested in DPI formulations, lactose is still the only excipient approved by the FDA in DPI formulations of APIs. At the present, the focus is to engineer carrier particles with modified physicochemical properties to promote the aerosolization of APIs to penetrate the deeper regions of the lungs [16].

The use of leucine has previously been shown to improve the aerosolization performance of several drugs from DPIs [17–19] because it reduces the inter-particulate adhesive forces and API aerodynamic particle size due to its surfactant behavior [20]. For example, leucine has been proven beneficial on Naringin dry powder as it has enhanced aerosol performance while improving the activity of cystic fibrosis airway epithelial cells [21].

It is recognized that lactose particles classified with being more elongated, being more irregular in shape, and having a rougher surface are exponentially preferred [14]. Consequently, the epitome of this overall study begins with using lactose monohydrate as a carrier of salbutamol sulfate with increasing leucine concentrations to manufacture an agglomerate spray-dried lactose-leucine system that will enhance the performance of salbutamol sulfate from DPI formulations. On the basis of this, the aim of the current research work was to investigate the effect of leucine on the performance of spray-dried lactose-leucine in DPI containing salbutamol sulfate.

## Materials and methods

### Materials

α-Lactose monohydrate (DFE Pharma, Gosh, Germany, purity 99 + %; lactose is one of the main excipients approved to be used in DPI formulation), salbutamol sulfate (L.B. Bohle, Germany; purity 99%), and _L_-leucine (Acros Organics, Geel, Belgium, purity 99 + %; it is used as anti-adherent and lubricant) were used in their purest form. Monobasic potassium phosphate (Fisher Scientific, UK) was used for the preparation of the mobile phase for high-pressure liquid chromatography (HPLC). All solvents (methanol, ethanol, and hydrochloric acid) were purchased from VWR International Ltd. (Leighton Buzzard, UK) and were of HPLC grade.

### Spray-drying

Spray-drying was conducted using the Mini Spray Dryer B-290 from Buchi (Flawil, Switzerland) equipped with a dehumidifier (Dehumidifier B-296), an inert loop (Inert Loop B-295), and an outlet filter. The parameters associated with the procedure were as follows: inlet temperature of 220 °C, aspirator set to 100%, pump rate set to 5%, and a flow rate of 22% designed for a closed environment with the use of nitrogen (N_2_) gas.

Each 100 mL of the solution for spray-drying contained different concentrations of leucine (0.1, 0.5, 1, 5, and 10 g) and 60 g of lactose. That means the percentage of leucine in each solution was 0.1, 0.5, 1, 5, and 10% *w*/*v*, respectively. Both leucine and lactose were dissolved in distilled water while heating the solution to 75 °C with a stirring speed of 120 rpm. The final solutions were spray-dried under the conditions mentioned above.

### Sieving

A Retsch AS 200 Digit Analytical Sieve Shaker (Hoan, Germany) was used to separate particle sizes within the 63–90-μm range (this is the standard particle size range for carriers that are used in DPI formulations). The collection pan was placed at the bottom, which was then followed by the 63-μm sieving pan, and finishing with the 90-μm sieving pan. The spray-dried particles were placed on top of the 90-μm sieving pan where sieving was performed for 30 min with an amplitude of 100 for each of the carriers. Particles that fell within the 63–90-μm range were collected in glass vials, sealed, and stored in an air-conditioned laboratory at a set temperature of 20 °C and a relative humidity (RH) of 50% for future use within this study.

### Particle size distribution analysis

Particle size distribution analysis was conducted using a laser diffraction particle size analyzer. The laser diffraction particle size analyzer (Sympatec Ltd., UK) is equipped with the HELOS sensor and Windox software and was used in combination with the RODOS (dry) system and Cuvette (wet) system; the wet system used absolute ethanol, a stirring speed of 1200 rpm, and 20 s of sonication. Detecting the particles was done using the R3 and R5 lenses, which have a particle size detection range of 0.5–175 and 0.5–875 μm, respectively.

The span of size distribution was calculated by Eq. 1:

1 $$ \mathrm{Span}=\left({\mathrm{D}}_{90\%}-{\mathrm{D}}_{10\%}\right)/{\mathrm{D}}_{50\%} $$

where D_90%_, D_50%_, and D_10%_ refer to the particle size (in μm) of 90, 50, and 10% of the cumulative particle size distribution, respectively.

### Preparation of DPI formulations

To the stored 63–90-μm sieved carriers, salbutamol sulfate (SS) was added such that a final ratio of 67.5:1 (Carrier: SS) was obtained. To prepare the final formulation, 1.35 g of each carrier was mixed with 20 mg SS using a Turbula blender (Type T2F, Junkermattstrasse, Switzerland) where each of the formulations was subjected to 30 min of blending at a speed of 72 rpm. The amount of formulation blend in each capsule was adjusted to 33 ± 1 mg. All filled capsules were stored for 24 h to decrease electrostatic forces prior to them being used in the deposition study. This 33 mg corresponds to a theoretical dosage of 482 ± 1.5 μg of SS per single unit.

### Differential scanning calorimetry analysis

To perform thermal analysis, Perkin Elmer’s (Shelton, Connecticut, USA) differential scanning calorimetry (DSC) 4000 equipped with a standard single-furnace was used. A known protocol was used [22], which includes using a temperature range of 25–300 °C (this range can cover all thermal events associated with lactose) while increasing the temperature at a rate of 10 °C/min. All samples were run under N_2_ gas, which allows for the samples to be purged, at a rate of 50 mL/min. Each of the samples was accurately weighed, where the mass ranged from 4 to 5 mg per sample on aluminum pans and sealed with an aluminum cap. Calibration was completed through the use of indium and zinc prior to any DSC run. All DSC traces were analyzed in terms of melting point and enthalpy with the accompanied Pyris Series software.

### Powder X-ray diffraction

Determining particle solid state was aided by the use of Siemens’ Diffractometer D5000 (Munich, Germany), and the implementation of a revised methodology of Kaialy et al. [23], where 200 mg of each carrier was placed on a holder such that a leveled surface was obtained when observed in comparison to the pan and diffractometer. The holder was then placed on the diffractometer in a manner where analysis at a specific angle was possible. The sample was exposed to CuК_α_ X-rays with a voltage of 40 kV and a current of 30 mA while being scanned from 5° to 50° on the 2θ plane at a scanning rate of 0.1 increments per second.

### Fourier transform infrared spectroscopy

To evaluate any interaction between the carriers and leucine at the molecular level, Perkin Elmer Spectrum One FT-IR Spectrometer (Shelton, Connecticut, USA) equipped with a universal ATR sampling accessory was used. Prior to analysis, methanol was used to clean the areas susceptible to leftover debris, after which a few milligrams of each of the carriers were used with a pressure of 100 bar. Each of the samples was scanned three times over a range of 4000 to 500 cm^−1^ to obtain spectra with appropriate resolution; no sample preparation was needed as the samples were analyzed as is.

### Scanning electron microscope

Electron micrographs were obtained using the JMS-820 scanning microscope (Freising, Germany) with a voltage of 4 kV as such approach allowed for the evaluation and characterization of particle morphology, size, shape, and presence or absence of agglomerates [24, 25]. Prior to taking images, each of the carriers was placed on double-sided carbon tape where the samples were coated for 5 min, under vacuum (in a closed system), with gold in an argon atmosphere to prevent their melting under the examination. To investigate the morphology of the carriers, different magnifications (refer to SEM images) were employed.

### Homogeneity assessment

To assess the uniformity of salbutamol sulfate in each of the formulations, 10 different samples (10–12 mg) were taken from each of the formulations in an ordered fashion; eight out of the 10 simulated a circle, while the ninth and tenth samples were directly obtained from the middle. The selected samples were dissolved in 100 mL of distilled water before HPLC analysis was completed, as explained below. The percentage potency and CV% of each formulation were calculated.

### Deposition study

A Multi-Stage Liquid Impinger (MSLI), equipped with a USP induction port (Copley Scientific in Nottingham, UK), was used alongside the critical flow controller (Copley TPK) and a high-capacity pump (Copley HCP5) allowing for a 4-kPa pressure drop to be observed. The MSLI has the capacity to filter particles, allowing for cutoff diameters to be taken into account in each of the individual stages when it is operated with a known flow rate. Calculating the cutoff diameter at a different flow rate is determined using Eq. 2 [26]:

2 $$ {\boldsymbol{D}}_{50,\boldsymbol{Q}}={\boldsymbol{D}}_{50,\boldsymbol{Q}\boldsymbol{N}}\sqrt{\raisebox{1ex}{${\boldsymbol{Q}}_{\boldsymbol{N}}$}\!\left/ \!\raisebox{-1ex}{$\boldsymbol{Q}$}\right.} $$

where *D*_50,*Q*_ refers to the cutoff diameter at the flow rate of *Q*, and *N* refers to the values obtained for each individual stage of the MSLI when the flow rate is 60 L/min. As a result, when such flow rate is used, the cutoff diameters for each of the individual stages become 13.00, 6.80, 3.10, and 1.70 μm, respectively. Furthermore, at the flow rate of 100 L/min, the cutoff diameters change and become 10.07, 5.27, 2.40, and 1.32 μm, respectively.

Each deposition study used 10 capsules per run, where every capsule was filled with 33 ± 1 mg of the carrier: SS (formulation) being investigated corresponding to the theoretical API dose of 482 ± 1.5 μg of salbutamol sulfate per capsule. The deposition test was repeated three times for each formulation, corresponding to 30 capsules per formulation.

In addition, specific parameters were employed for the analysis of the aerosolization performance of each formulation including the recovery dose (RD), emitted dose (ED), percent recovery, percent emission, impaction loss, mass median aerodynamic diameter (MMAD), geometric standard deviation (GSD), fine particle fraction (FPF), fine particle dose (FPD), drug loss (DL), dispersibility (DS), and effective inhalation index (EI).

Additionally, RD is defined as the amount of drug (in μg) recovered from the inhaler, induction port (IP), mouthpiece (M), and stages 1–5 (S1–S5); ED as the amount of drug (in μg) recovered from IP and S1–S5; percent recovery as the ratio of RD to the theoretical dose (482 ± 1.5 μg); percent emission as the ratio of ED to RD; impaction loss as the mass fraction of drug in IP and S1 to RD (IP + S1: RD); MMAD as the logarithmic function of the cutoff diameter to the corresponding concentrations of particles found within each stage of the MSLI; GSD as the square root of the 84th and 15th percentiles; FPF as the ratio between FPD to RD; FPD as the sum of drug (in μg) from S3–5; DL as the ratio of the amount of salbutamol sulfate recovered from capsules, mouthpiece, and inhaler to RD [(capsules + (I + M)): RD]; and DS as the ratio of FPD to ED (FPD:ED).

Moreover, to determine the effective inhalation index (EI) of each formulation, Eq. 3 was implemented where EI refers to the effective inhalation index, and EM and FPF are the percent emission and fine particle fraction [27].

3 $$ \boldsymbol{EI}=\sqrt{\left(\boldsymbol{EM}+\boldsymbol{FPF}\right)} $$

All the deposition studies were conducted in an air-conditioned laboratory where the temperature was 20 °C and the relative humidity (RH) was 50%.

### High-pressure liquid chromatography

To quantify salbutamol sulfate in the homogeneity test or deposition study, a mobile phase containing 95% (*v*/*v*) of 25 mM potassium phosphate (monobasic) pH 3.0 and 5% (*v*/*v*) of methanol was used. The flow rate of the mobile phase through the HPLC column was 1.5 mL/min with a total run time of 25 min per injection set at a wavelength of 225 nm yielding a retention time of 12 min. To adjust the pH to 3.0, a 1 M HCl solution was used while stirring at 180 rpm, after which the mobile phase was filtered and degassed using a Fisher Scientific (Leicestershire, England) 0.22-μm filter before its use.

HPLC was executed via the Agilent 1100 series HPLC system (Santa Clara, California, USA) where a degasser (G1322A), binary pump (G1312A), variable wavelength detector (VWD G1314A), column thermostat (G1316A), and thermostatic autosampler (ALS G1329A) coupled with the Waters Spherisorb 5 μm ODS2 4.6 × 150 mm analytical column (Milford, Massachusetts, USA). Likewise, internal standards of varying salbutamol sulfate concentrations (0.00. 0.50, 2.50, and 5.00 μg/mL, respectively) were used to calibrate and normalize the results.

## Results and discussion

### Particle size analysis

**Fig. 1:**
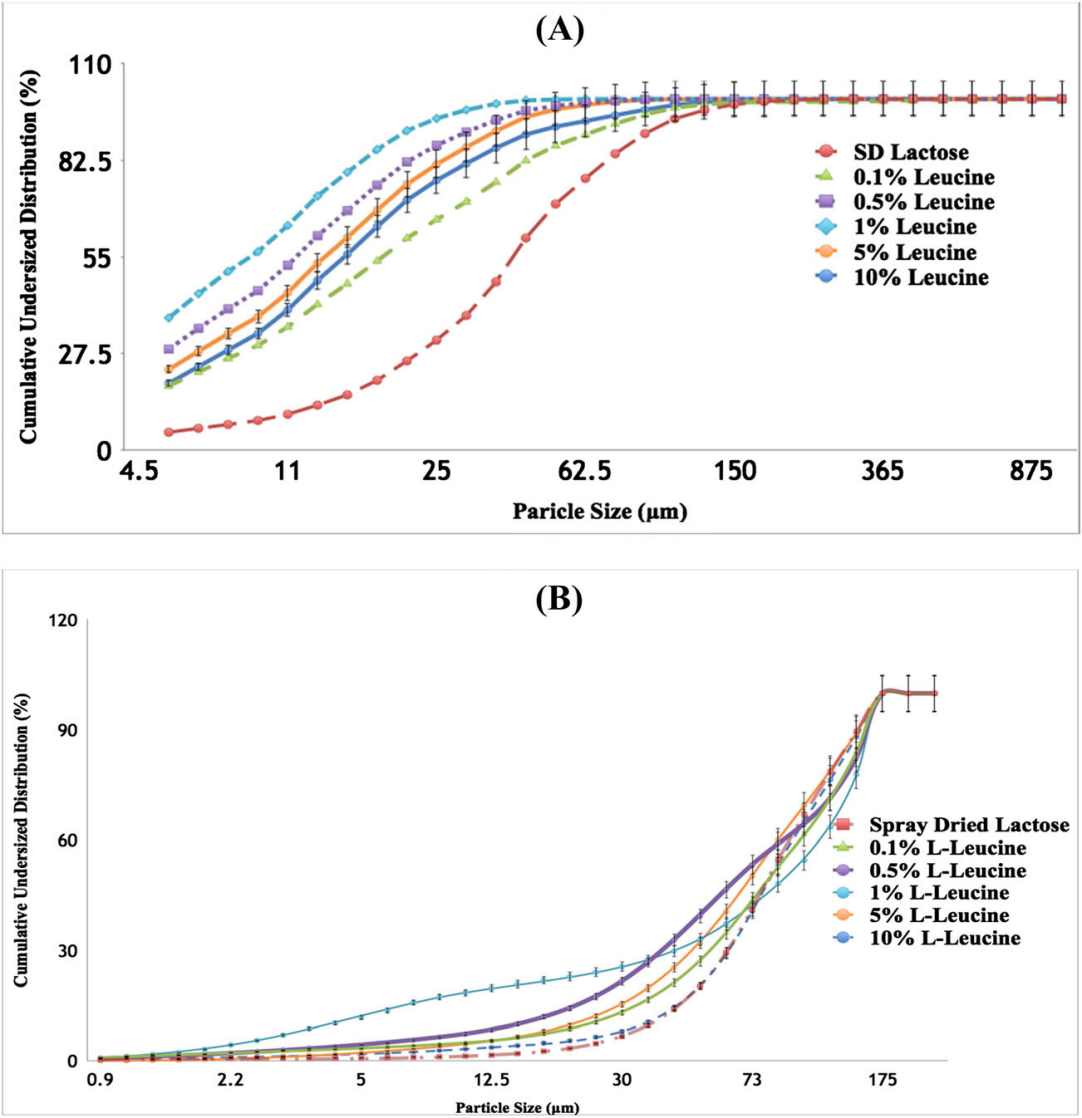
Particle size distribution (PSD) diagrams of each carrier when using the **a** RODOS dry system and when using the **b** CUVETTE wet system; the carriers used were spray-dried lactose monohydrate containing 0, 0.1, 0.5, 1, 5, and 10% _L_-leucine

Figure 1 shows two cumulative particle size distribution (PSD) diagrams that illustrate each of the carrier’s PSD when using the (A) dry system and when using the (B) wet system. Moreover, given the use of the Mini Spray Dryer B-290, it was expected to obtain particle sizes that were below that of the 63–90-μm range (Fig. 1a), given that the spray-drying process produces particles below such range [28].

Likewise, Fig. 1b shows that the particle sizes of each of the carriers, when using the wet system, fell within the 63–90-μm range. The known occurrence of agglomeration was exploited in such a manner that allowed for it to be used as a carrier within this overall study. Such focus offered the opportunity to investigate the agglomerates on the basis of parameters that are used in the characterization and analysis of single particles. Particle size measurement in the dry system was able to break the aggregates of spray-dried particles (due to applying a pressure of 3 bar during the measurement) whereas in the wet system the spray-dried particles stayed as aggregates, although the ultrasound was applied during the measurement.

Relatedly, Table 1 highlights each of the carrier’s distinct characteristics such as volume mean diameter (VMD) and span of the RODOS dry system and the CUVETTE wet system comparing each characteristic side by side. Table 1 shows that all of the carriers experienced a significant difference in their VMDs when comparing each system to one another. For the dry system, the VMD ranged from 8.39 ± 0.40 to 37.97 ± 0.08 μm whereas the range for the wet system was 79.31 ± 2.19 to 87.95 ± 1.91 μm due to the presence of aggregated particles. The dry system experienced a particle diameter range of 1.56 ± 0.05 μm (D_10%_) to 75.46 ± 7.22 μm (D_90%_) whereas the particle diameter range for the wet system fell between 20.77 ± 11.02 μm (D_10%_) and 147.87 ± 170.11 μm (D_90%_). Due to the aggregation of particles, samples that were measured through the wet system showed smaller span values (narrower distribution) compared to that of the dry system, coinciding with the possibility of the dry system containing mixtures of aggregated and de-aggregated spray-dried particles during the measurement (Table 1).

**Table 1:** Particle analysis of spray-dried lactose monohydrate and spray-dried lactose monohydrate-leucine where the concentration of leucine was 0.1, 0.5, 1, 5, and 10% showing the volume mean diameter (VMD) and span when using the RODOS dry system vs. the CUVETTE wet system (mean + standard deviation)

Formulation	VMD (μm) dry system	VMD (μm) wet system	Span dry system	Span wet system
Spray-dried lactose	37.97 ± 0.08	87.14 ± 0.35	2.12 ± 0.09	1.36 ± 0.01
0.1% _L_-leucine	23.95 ± 5.35	87.47 ± 4.94	3.61 ± 0.11	1.61 ± 0.14
0.5% _L_-leucine	11.50 ± 0.28	79.68 ± 1.78	2.67 ± 0.13	2.14 ± 0.10
1% _L_-leucine	8.39 ± 0.40	86.88 ± 0.84	2.57 ± 0.06	1.73 ± 0.03
5% _L_-leucine	13.58 ± 0.20	79.31 ± 2.19	2.62 ± 0.12	1.74 ± 0.08
10% _L_-leucine	17.47 ± 0.63	87.95 ± 1.91	3.10 ± 0.23	1.40 ± 0.02

Such outcomes, then, allowed for the carriers to be implemented and further studied to determine their physicochemical properties and particle morphology given that they underwent spray-drying, known to alter such characteristics, while also introducing _L_-leucine as an excipient. VMD (obtained via wet method) of the formulation after mixing for 30 min with salbutamol sulfate showed that the mixing process was unable to break down the agglomerates as the VMD was similar to the VMD of particles before mixing. For example, the VMD of formulations containing 0.1 and 10% leucine after 30 min of mixing with SS was 88.0 ± 9.62 and 71.97 ± 0.16 μm, respectively.

**Fig. 2:**
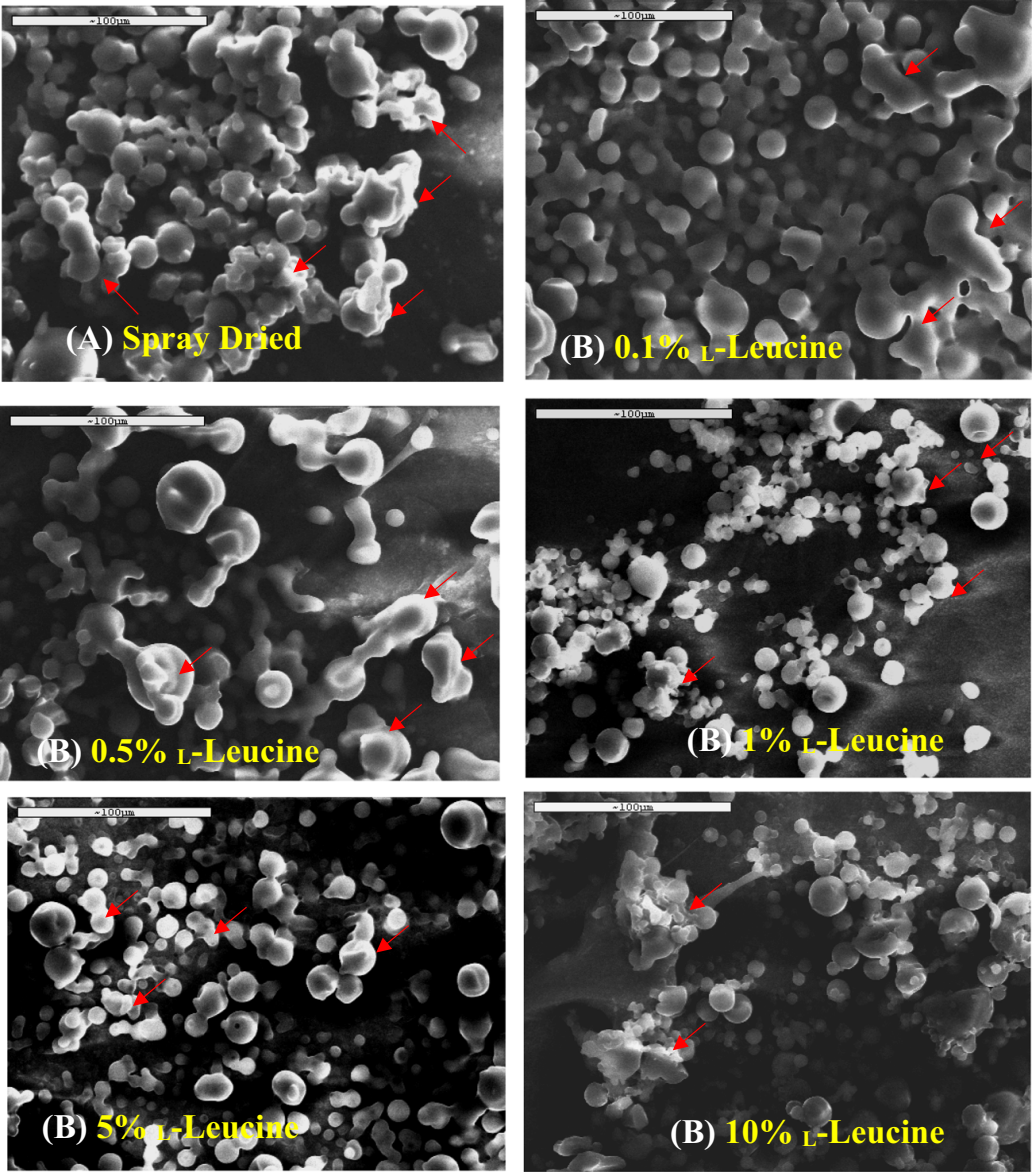
SEM images of **a** spray-dried lactose monohydrate, spray-dried lactose containing-leucine where leucine concentrations were **b** 0.1%, **c** 0.5%, **d** 1%, **e** 5%, and **f** 10%

Figure 2 highlights the electron micrographs of each of the carriers making it evident that all of the formulations, with respect to their carrier, experienced some agglomeration giving way to their larger particle size, thereby supporting the results presented in Fig. 1 and Table 1. Moreover, the SEM micrographs also indicate, and account for, each of the carrier’s morphology as they show that all of them contain spherical particles with some agglomerates, particularly in the cases of 0.5% leucine (some of these agglomerated particles for each formulation are shown by red arrows). Such irregularity has previously been shown to be more effective in the delivery of salbutamol sulfate when compared to particles that are classified as being more spherical and regular in shape [22]. The morphology of the spray-dried lactose, with increasing concentrations of leucine, is supported by data published by Aquino et al. [29], where they showed that more irregular and corrugated particles were obtained in the presence of high concentrations of leucine. Generally, corrugated particles disperse better than spherical ones as this kind of particle reduces contact areas and decreases inter-particulate cohesion. Therefore, it was expected that the formulation composition of the leucine carrier would result with an enhanced aerosolization performance, when compared to the carrier without leucine, which, in essence, would deliver salbutamol sulfate more poorly.

### Solid-state characterization of spray-dried samples

**Fig. 3:**
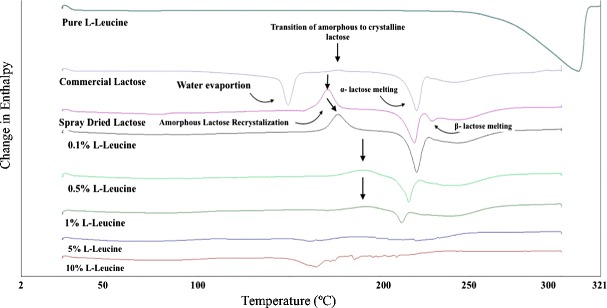
DSC thermal peaks of _L_-leucine, commercial lactose monohydrate, spray-dried lactose monohydrate-leucine where the concentration of leucine were 0, 0.1, 0.5, 1, 5, and 10% *w*/*v* (where an exothermic peak would point up and an endothermic peak would point down)

Figure 3 shows DSC traces of _L_-leucine, original lactose monohydrate, and spray-dried lactose containing 0, 0.1, 0.5, 1, 5, and 10% _L_-leucine indicating where water evaporation, amorphous lactose recrystallization (H_c_), α-lactose melting (H_α_), and β-lactose melting (H_β_) took place. It is obvious from the figure that commercial lactose monohydrate shows an endothermic peak around 149 °C, which corresponds to the evaporation of water, followed by an exothermic peak around 171 °C, indicating the amorphous state in the sample; moreover, the endothermic peak around 220 °C corresponds to the melting of α-lactose [30] whereas any peak around 238 °C is an indication of β-lactose in the sample.

Pure _L_-leucine was also tested to determine whether or not any thermal events arose between the 25–300 °C range, which would rule out whether such events were due to the presence of leucine or not. No thermal events were seen within the range where lactose thermal events occurred and the endothermic peak around 300 °C corresponds to the melting of leucine. Spray-dried lactose showed three main thermal events with the first being an exothermic peak around 170 °C, attributed to the recrystallization of amorphous lactose to both α-lactose and β-lactose, which was then followed by the melting of α-lactose at around 220 °C; furthermore, the third endothermic peak around 238 °C was an indication of β-lactose in the sample. Moreover, spray-dried lactose did not show any DSC traces for the water evaporation which was a similar pattern that was observed for spray-dried lactose containing 0.1, 0.5, and 1% leucine, but with different intensities when compared to the spray-dried carrier with no leucine. Spray-dried formulations containing 5 and 10% _L_-leucine did not show any sharp or obvious peaks for water evaporation, the transition of amorphous lactose to crystalline lactose, and melting of lactose. On the basis of this information, all spray-dried carriers were considered to be in their amorphous state as the data that was collected indicates given that a definite crystalline structure was not present prior to their analysis which would have been depicted through the emergence of the amorphous lactose recrystallization enthalpy. Neither a recrystallization nor a melting peak was observed in the 5 and 10% _L_-leucine carrier, which serves as an indicator of their higher stability against recrystallization. Such results follow similar patterns that have been presented elsewhere [31–34] where amorphous drug carriers were formulated in such a way as to increase amorphous stability. To make sure spray-dried lactose was in the amorphous state, a more reliable technique (PXRD) was used.

**Fig. 4:**
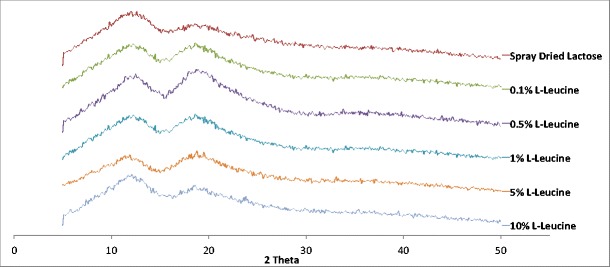
X-ray diffraction patterns of spray-dried lactose monohydrate and spray-dried lactose monohydrate-leucine where the concentrations of leucine were 0.1, 0.5, 1, 5, and 10%

Figure 4 contains the x-ray diffraction peaks for spray-dried lactose monohydrate containing 0, 0.1, 0.5, 1, 5, and 10% _L_-leucine, which provides an insight into the polymorphic state of each of the carriers. A carrier’s morphology plays an integral role in the drug delivery process that dictates whether a formulation is deemed effective in the delivery of the API of interest [30].

Looking at Fig. 4 more closely, it becomes evident that all of the carriers, from each of the formulations, were classified as being in their amorphous state given the absence of peaks (halo structure). In addition, all of the carriers showed two distinct peaks each (2θ = 12.2° and 18.6°) that are broad and distributed over a wide range of degrees on the 2θ plane, which also characterizes them as being of amorphous state. Likewise, given that all of the carriers exhibited irregular diffraction of electromagnetic radiation when compared to pure _L_-leucine (XRD not shown), it correspondingly catalogs them as amorphous as well [35].

**Fig. 5:**
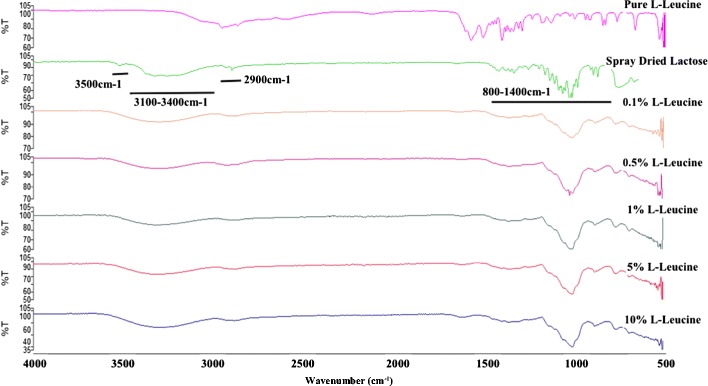
FT-IR spectra of pure _L_-leucine, spray-dried lactose monohydrate, and spray-dried lactose monohydrate-leucine where the concentrations of leucine were 0.1, 0.5, 1, 5, and 10% highlighting the areas that are eliminated or broadened as the concentration of _L_-leucine increases while also showing where the amorphous, α-lactose, and β-lactose peaks are to be found

To further assess the solid state of each carrier within this study and identify any interaction between lactose and leucine at the molecular level, FT-IR spectroscopy was implemented with the understanding that amorphous lactose displays a distinct frequency at 1260 and at 900 cm^−1^, α-lactose monohydrate at 920 cm^−1^, and β-lactose at 950 cm^−1^ [30]. Figure 5 presents the results for the FT-IR spectra of _L_-leucine, spray-dried lactose monohydrate with its different concentrations of _L_-leucine (0, 0.1, 1, 5, and 10%), and further supports the fact that the carriers are in their amorphous state as the aforementioned peaks were present. In addition, Fig. 5 also reveals that, with the increasing concentration of _L_-leucine, each formulation underwent a phenomenon known as Fermi resonance where a shift in the vibrational energy causes the spectra to have a change in its intensity and resolution [36, 37].

Such variation within the spectra explains why the frequencies that are associated to key functional groups like aromatic C-H, alkanes, aldehydes, hydroxyl, carbonyl, ethers, and primary amines (which have frequencies at 2900, 3100–3400, 800–1400, and 3500 cm^−1^) become broadened or eliminated completely.

### In vitro analysis of DPI formulations

#### Salbutamol sulfate assessment

**Fig. 6:**
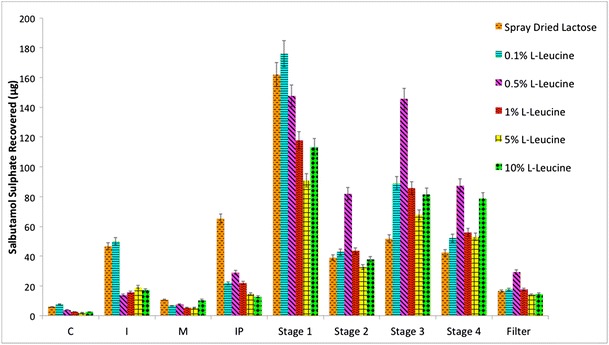
Aerosolization performance of each of the formulations (spray-dried lactose monohydrate and spray-dried lactose monohydrate-leucine where the concentrations of leucine were 0.1, 0.5, 1, 5, and 10% highlighting the amount of SS recovered (percent recovered)

Performance of the drug delivery profile of salbutamol sulfate, with respect to each of the formulations within this overall study, is defined in Fig. 6 where the amount of salbutamol sulfate recovered from each individual section within the MSLI is looked with a narrower focus: capsules (C), inhaler (I), mouthpiece (M), induction port (IP), stage 1, stage 2, stage 3, stage 4, and filter (stage 5). All of the formulations experienced minimal salbutamol sulfate deposits in the capsules with 5 and 10% _L_-leucine having the least amount after their actuation due to the lubrication effect of leucine, which makes particles flow more easily from the capsule to the inhaler device (Cyclohaler). The lubrication effect of the spray-dried leucine has been reported where increasing amounts of _L_-leucine show good lubricating properties [38]. As particles maneuver through the respiratory tract, spray-dried lactose monohydrate along with 0.1% _L_-leucine experienced the highest amounts of salbutamol sulfate (43.63 ± 23.48 and 49.89 ± 27.80 μg, respectively) in the inhaler device when compared to the concentrations above 0.5% _L_-leucine which experienced the least amount at 13.79 ± 11.47 μg; the lubrication effect of leucine can also be observed here, as described previously. Furthermore, all of the formulations showed about the same amount of salbutamol sulfate in the mouthpiece (Fig. 6) but begin to differ at the IP as spray-dried lactose monohydrate had the highest amount (65.24 ± 4.26 μg) when compared to the other formulations, which had a range of 12.66 ± 5.66 to 29.02 ± 18.56 μg.

Moreover, 0.1% _L_-leucine had the highest salbutamol sulfate recovered from within stage 1 (176.06 ± 50.94 μg), but where it began to change was with stage 2 onward as 0.5% _L_-leucine experienced the highest salbutamol sulfate amounts in stage 2, stage 3, stage 4, and filter (81.89 ± 50.20, 145.58 ± 88.08, 87.45 ± 48.49, and 29.25 ± 20.16 μg, respectively) indicative of it being the most successful at delivering salbutamol sulfate to the targeted area that correlates to the alveoli, found in the lower respiratory tract. In other words, the formulations ranked in the following order 0.5% _L_-leucine > 0.1% _L_-leucine > 1% _L_-leucine > spray-dried lactose monohydrate > 5% _L_-leucine > 10% _L_-leucine.

Table 2 shows the aerosolization performance and deposition data for all formulations studied. The authors in the present study were interested in looking at, namely, FPF (fine particle fraction), DL (drug loss), impaction loss (IL), and effective inhalation efficiency (EI). All of the formulations differed remarkably from one another with respect to DL and percent emission (Table 2) given that they all undertook a high number of actuations (*n* = 10) per run, with each being filled with a consistent weight of around 33 ± 1 mg. The table shows that the performance of DPI formulations containing spray-dried leucine is much better than when leucine was excluded from the formulation (the lowest drug loss belonged to spray-dried lactose containing 0.5% _L_-leucine).

**Table 2:** Recovered dose (RD), emitted dose (ED), percent recovery, percent emission, percent impact loss, mass median aerodynamic diameter (MMAD), geometric standard deviation (GSD), fine particle dose (FPD), fine particle fraction (FPF), drug loss (DL), dispersibility (DS), and effective inhalation index (EI) of salbutamol sulfate obtained from each of the different formulations (spray-dried lactose monohydrate and spray-dried lactose-leucine where the concentrations of leucine were 0.1, 0.5, 1, 5, and 10%)

Formulation	RD (μg)	ED (μg)	Recovery (%)	Emission (%)	Impact loss (%)	MMAD (μm)	GSD (μm)	FPD (μg)	FPF (%)	DL (%)	DS (%)	EI
SD lactose	434.09 ± 40.27	376.79 ± 19.65	90.25 ± 8.37	87.02 ± 3.79	52.49 ± 2.81	3.13 ± 0.15	2.18 ± 0.07	110.41 ± 4.77	25.51 ± 1.23	14.31 ± 4.12	29.31 ± 0.36	10.87 ± 0.22
0.1% _L_-leucine	455.47 ± 53.13	398.95 ± 29.95	94.69 ± 11.05	87.92 ± 4.89	43.46 ± 10.14	3.19 ± 0.09	2.09 ± 0.03	158.53 ± 39.73	34.99 ± 8.89	13.72 ± 5.34	39.62 ± 8.79	10.93 ± 0.39
0.5% _L_-leucine	542.26 ± 297.51	520.92 ± 281.30	112.74 ± 61.85	96.41 ± 1.23	34.61 ± 12.38	3.26 ± 0.05	2.09 ± 0.01	262.28 ± 156.60	47.11 ± 9.94	4.14 ± 1.52	48.94 ± 10.78	11.98 ± 0.37
1% _L_-leucine	363.63 ± 49.05	342.77 ± 51.17	75.60 ± 10.20	94.15 ± 1.54	37.60 ± 10.86	3.16 ± 0.13	2.10 ± 0.03	159.45 ± 14.31	44.33 ± 6.53	6.55 ± 1.53	47.12 ± 7.23	11.77 ± 0.26
5% _L_-leucine	297.32 ± 175.08	272.87 ± 179.66	61.81 ± 36.40	88.91 ± 7.43	35.95 ± 4.00	3.00 ± 0.18	2.13 ± 0.08	134.81 ± 91.41	43.08 ± 7.38	12.00 ± 8.18	48.26 ± 5.35	11.48 ± 0.63
10% _L_-leucine	366.52 ± 166.33	338.81 ± 149.50	76.20 ± 34.58	92.82 ± 2.34	38.74 ± 22.45	2.99 ± 0.23	2.12 ± 0.08	174.83 ± 121.38	44.50 ± 17.40	7.82 ± 2.26	48.27 ± 19.67	11.71 ± 0.66

Impact loss (IL) within the formulations varied from 34.61 ± 12.38%, attributed to 0.5% _L_-leucine, to 52.49 ± 2.81%, belonging to spray-dried lactose monohydrate. Such variation between the formulations could be attributed to their aerodynamic diameter given that impaction is a flow-dependent mechanism governed by particle size [39].

Effective inhalation index (EI) ranged from 10.87 ± 0.22 (spray-dried lactose monohydrate) to 11.98 ± 0.37 (0.5% _L_-leucine) aligning with other data suggesting that 0.5% _L_-leucine has a high-drug aerosolization efficiency.

DS and FPD also showed a variation among the formulations with ranges of 29.31 ± 0.36 to 48.94 ± 10.78% and 262.28 ± 156.60 to 110.41 ± 4.77 μg, respectively. Such variation was attributed to the formulation’s particle size given that the phenomenon of inertial impaction becomes prevalent for large particles [39].

When it came to MMAD and GSD, however, all of the formulations gave similar results with MMAD being 3.12 ± 0.10 μm and GSD being 2.12 ± 0.03 μm. Such results mean that all of the SS was, theoretically, delivered to the area of keen interest. It also correlates with obtaining a FPF of 100%, which was not the case, as other factors came into play like impaction onto the upper respiratory tract where particles greater than or equal to 10 μm are removed by the mucociliary escalator and subsequently swallowed [40, 41].

The results showed that spray-dried lactose-leucine (containing 0.5% _L_-leucine) exhibited the highest FPF of 47.11 ± 9.94% suggesting that such formulation was the most efficient at delivering the most SS to the lower respiratory tract. This is because of the correlation that is seen between FPF and amount of SS delivered; that is to say, when FPF increases, the expected amount of SS that is delivered to the lower respiratory tract also increases [42]. Such values, when compared to those obtained by Kaialy et al. [23] (FPF of 44.85 ± 1.76%) and Kaialy and Nokhodchi [43] (FPF of 46.9 ± 3.6%), prove to be an increase in the efficacy of salbutamol sulfate’s aerosolization performance. This formulation also had the highest percent emission of 96.41 ± 1.23%, when compared to the other formulations (Table 2), suggesting that SS was able to detach itself from the carrier easier when compared to the other formulations. This means that optimal physicochemical properties were attained such that a complementary system emerged between SS and the 0.5% _L_-leucine carrier. On the other hand, spray-dried lactose monohydrate showed the lowest percent emission (87.02 ± 3.79%) and consequently the lowest FPF (25.51 ± 1.23%). Such results infer that SS had a more difficult time detaching itself from the spray-dried lactose monohydrate carrier during inhalation when compared to 0.5% _L_-leucine.

#### Homogeneity assessment

Assessing the homogeneity of each of the formulations was an essential phase of this overall study given that a uniform formulation will give rise to a more effective drug delivery profile with a consistent dose to the patient. Table 3 eludes the homogeneity profile of each of the formulations (spray-dried lactose monohydrate, samples spray-dried with 0.1, 0.5, 1, 5, and 10% _L_-leucine) under investigation showing the potency of each and also presents the percent content homogeneity, which is expressed as the percent coefficient of variation (%CV), of each of the aforementioned formulations. The drug content of all formulations was within 75–125%, and the smallest %CV of 5.48% belonged to 0.5% _L_-leucine, which was the formulation that showed the best aerosolization performance. Such results indicate that 0.5% _L_-leucine had the best salbutamol sulfate content homogeneity among all of the formulations followed by 0.1% _L_-leucine with a %CV of 7.15%. In addition, the results showed that it is a bit difficult to obtain a very low CV% for DPI formulation containing salbutamol sulfate in the DPI formulation studied in the current research. This should be investigated more in the future ongoing research.

**Table 3:** Content homogeneity of spray-dried lactose monohydrate, 0.1% _L_-leucine, 0.5% _L_-leucine, 1% _L_-leucine, 5% _L_-leucine, and 10% _L_-leucine expressed as the percent coefficient of variation (%CV)

Formulation	Potency	%CV
Spray-dried lactose	91.36 ± 9.40	10.29
0.1% _L_-leucine	83.62 ± 5.98	7.15
0.5% _L_-leucine	102.66 ± 5.62	5.48
1% _L_-leucine	94.72 ± 16.97	17.92
5% _L_-leucine	89.71 ± 11.87	13.23
10% _L_-leucine	89.98 ± 7.78	8.65

## Conclusion

_L_-Leucine’s addition allowed for the emergence of a more effective aerosolization performance as it assisted in the altering of lactose monohydrate’s physicochemical properties in such a way as to provide a two-fold increase in the fine particle fraction that equates to an increase in salbutamol sulfate’s concentration in the lower respiratory tract. In addition, through this study, it has been proven that _L_-leucine improves the stability of amorphous spray-dried lactose as was evident from DSC traces while simultaneously providing a lubrication effect for the formulations that were studied.
